# Silicon-induced photosynthetic adaptations in common buckwheat under salt stress revealed by prompt chlorophyll *a* fluorescence analysis

**DOI:** 10.1038/s41598-025-04159-1

**Published:** 2025-06-02

**Authors:** Md. Rakib Hossain Raihan, Michal Antala, Marcin Stróżecki, Md. Intesaful Haque, Mirza Hasanuzzaman, Radosław Juszczak, Anshu Rastogi

**Affiliations:** 1 Laboratory of Bioclimatology, Department of Ecology and Environmental Protection, Poznań University of Life Sciences, Poznań 60-649, Poland; 2 Department of Agronomy, Faculty of Agriculture, Sher-e-Bangla Agricultural University, Sher-e-Bangla Nagar, Dhaka-1207, Bangladesh; 3 Pioneer Center Land-CRAFT, Department of Agroecology, Aarhus University, Aarhus, Denmark

**Keywords:** Salinity, Ionic stress, Oxidative stress, OJIP transient, JIP test, Photosynthesis, Photosynthesis, Plant physiology, Plant stress responses

## Abstract

This study aimed at investigating the protective role of silicon (Si) in mitigating salt-induced damage in common buckwheat plants (*Fagopyrum esculentum* cv. Smuga). Twenty one-day-old seedlings were subjected to salt stress by irrigating 50 mM sodium chloride solutions for seven days, with or without Si (two foliar applications with 1 mM sodium metasilicate nonahydrate). Salt stress significantly altered the chlorophyll *a* fluorescence transient (OJIP) curve, disrupting energy flow and electron transport in photosystem II (PSII), as reflected in the O-J, J-I, and I-P phases, along with the emergence of a positive K-band indicating damage to the oxygen-evolving complex (OEC). Silicon application mitigated these effects, stabilizing the OEC and thylakoid membrane integrity while improving JIP test parameters and reducing excessive energy absorption, dissipation, and unregulated energy loss per reaction center. Silicon-treated plants under salt stress exhibited enhanced photochemical quenching, reduced regulatory energy dissipation, and decreased photosystem I (PSI) over-reduction. A significant increase in open PSI centers was observed, improving the balance and functionality between PSI and photosystem II. The application of Si resulted in significant photosynthetic improvements, which were also paired with enhanced morphological traits, such as increased root length and leaf thickness in saline conditions. Overall, findings indicate that exogenous Si helps to reduce salt-induced stress by enhancing photosynthetic efficiency in plants, positioning it as a promising strategy for improving crop performance in saline environments.

**Supplementary Information:**

The online version contains supplementary material available at 10.1038/s41598-025-04159-1.

## Introduction

The global expansion of saline-prone areas, driven by excessive fertilizer use, irrigation with saline water, and climate change, poses a growing threat. Among the abiotic stresses, salinity is particularly critical due to its widespread prevalence and capacity to impair plant growth and development, ultimately reducing yields^1^. Salinity reduces osmotic potential, making water absorption by plants more challenging and inducing osmotic stress. High salinity leads to ionic imbalances as sodium ion (Na⁺) disrupts ionic homeostasis by displacing potassium (K⁺), raising the Na⁺/K⁺ ratio, and impairing cellular processes essential for plant growth^2,3^. One of the common consequences of salinity is oxidative stress due to the overproduction of reactive oxygen species (ROS), which damage cellular membranes, accelerate tissue necrosis, and reduce plant vigor^4,5^. High salinity restricts stomatal conductance, limiting carbon dioxide availability, and accelerating chlorophyll (Chl) degradation, which reduces light-capturing efficiency. Salinity also compromises the structural and functional integrity of photosystem II (PSII), inhibiting photochemical activity, electron transport chains (ETC), and carbon assimilation while exacerbating oxidative damage in chloroplasts^6,7^. Consequently, salinity significantly reduces photosynthetic capacity, leading to stunted growth, lower biomass accumulation, and reduced crop yields^8^.

Ameliorating the effects of salinity on crops is critical for sustaining agricultural productivity. Enhancing salt tolerance through mechanisms such as improved ion homeostasis, stabilization of chloroplast function, and ROS detoxification can protect photosynthesis and ensure plant growth under saline conditions. One promising approach involves silicon (Si), an element increasingly recognized for enhancing plant resilience to environmental stressors^9^. Although it is not essential for plants Si is classified as a beneficial and quasi-essential element, acknowledging its significant contributions to plant health and stress resilience^10,11^. Silicon supplementation has shown considerable benefits under adverse conditions by improving plant growth and enhancing stress tolerance. Silicon enhances plant health by strengthening structural rigidity, improving ion balance, activating antioxidant defense systems, and regulating genes^12^. These mechanisms help plants withstand challenges like salinity, drought, high temperatures, and heavy metal toxicity^13,14^. Under salinity stress, Si enhances photosynthesis by stabilizing Chl, improving stomatal conductance, and protecting PSII efficiency from ROS damage^9^.

Buckwheat (*Fagopyrum esculentum*), belongs to the Polygonaceae family and is cultivated globally for its nutritional and medicinal value. Rich in rutin, polyphenols, dietary fibers, and essential amino acids like lysine, it supports health by preventing chronic conditions such as obesity, hypertension, and cardiovascular diseases, while also serving as a gluten-free option for celiac patients^15–17^. It can thrive in harsh environments, such as high-altitude mountainous regions and arid or semi-arid areas, characterized by intense ultraviolet radiation, and frequent droughts, which can limit its growth and yield^18^. While research has highlighted buckwheat’s adaptation to various stresses, limited studies have explored the specific impacts of salinity on its growth and productivity. This study offers new insights by examining the impact of Si on the photosynthetic performance of buckwheat, a crop that has received limited attention in this context. Unlike previous studies, our research incorporates comprehensive fluorescence-based analyses to elucidate the mechanisms by which Si mitigates salt stress in buckwheat. Therefore, understanding the role of Si in alleviating salt stress could provide valuable insights for enhancing crop resilience in challenging environments.

## Results

### Effect of foliar application silicon on morphological traits of buckwheat under NaCl stress

**Fig. 1:**
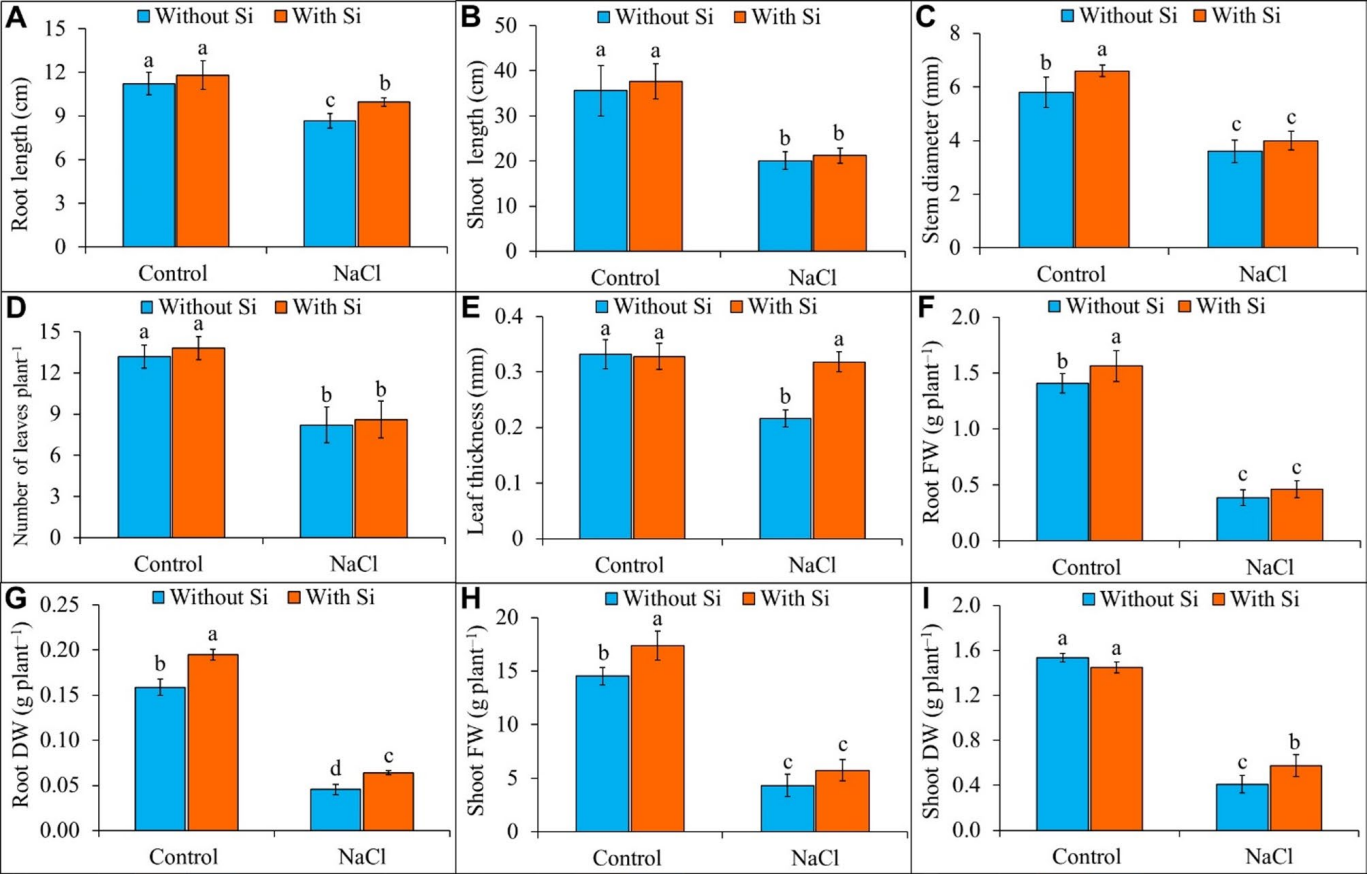
Effect of foliar application of silicon on the growth parameters, **(A)** root length, **(B)** shoot length, **(C)** stem diameter, **(D)** number of leaves per plant, **(E)** leaf thickness, **(F)** root fresh weight (FW), **(G)** root dry weight (DW), **(H)** shoot FW, and **(I)** shoot DW of buckwheat (*Fagopyrum esculentum* cv. Smuga) under NaCl stress. Data are presented as mean ± SD (*n* = 5); different letters indicate significant differences among treatment groups after applying a Tukey’s HSD test at *p* ≤ 0.05. Treatments: Control (no NaCl or Si application), Silicon (foliar application of 1 mM Si without NaCl), NaCl (50 mM NaCl treatment without Si application), and NaCl + Silicon (foliar application of Si under NaCl stress).

Salt stress caused a substantial reduction in key morphological traits, including root length, shoot length, stem diameter, number of leaves, leaf thickness, fresh weight, and dry weight in buckwheat plants. Exposure to NaCl resulted in a marked reduction of 23% in root length (Fig. 1A), 44% in shoot length (Fig. 1B), 38% in stem diameter (Fig. 1C), 38% in the number of leaves (Fig. 1D), 35% in the leaf thickness (Fig. 1E), 72% in root fresh weight (FW; Fig. 1F), 71% in root dry weight (DW; Fig. 1G), 70% in shoot FW (Fig. 1H), and 73% in shoot DW (Fig. 1I) compared to control plants. The foliar application of Si enhanced several morphological traits in plants under NaCl stress. However, under salt stress, Si application led to an increase of 15% in root length (Fig. 1A), 47% in leaf thickness (Fig. 1E), root DW in 41% (Fig. 1G), and shoot DW in 40% (Fig. 1I).

**Fig. 2:**
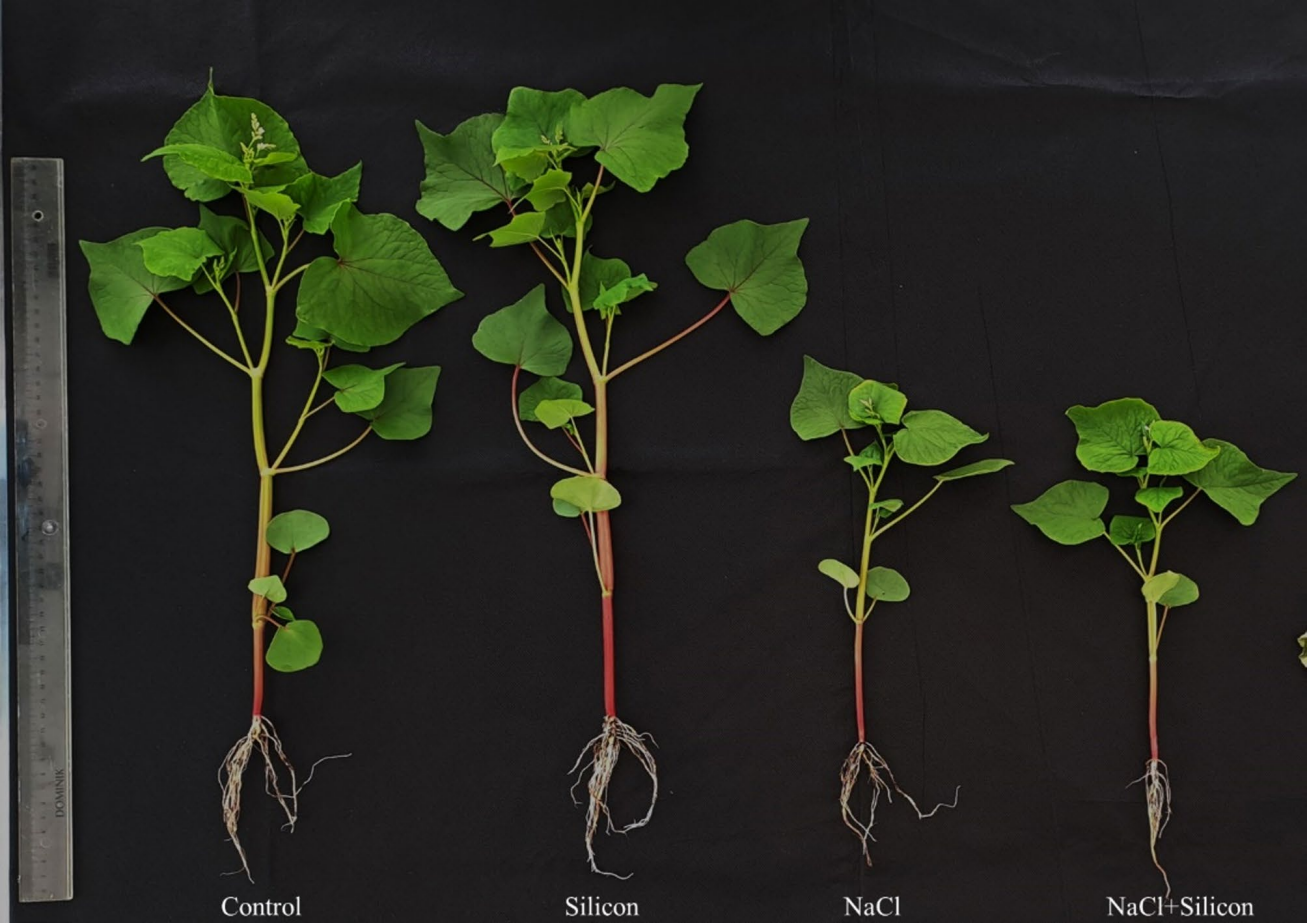
Morphological representation of buckwheat plants (*Fagopyrum esculentum* cv. Smuga) after 7 days of treatment exposure. Treatments: Control (no NaCl or Si application), Silicon (foliar application of 1 mM Si without NaCl), NaCl (50 mM NaCl treatment without Si application), and NaCl + Silicon (foliar application of Si under NaCl stress).

Each treatment group exhibited distinct morphological characteristics. Control plants (untreated with NaCl or Si) and those treated only with Si displayed a healthier appearance, while NaCl-treated plants showed signs of stress, including pale green leaves, and reduced height of the plants. However, the NaCl + Si treated plants demonstrated improved growth compared to the NaCl only, indicating partial recovery from salt stress (Fig. 2).

### Influence of silicon application on chlorophyll *a* fluorescence transient kinetics under salt stress

**Fig. 3:**
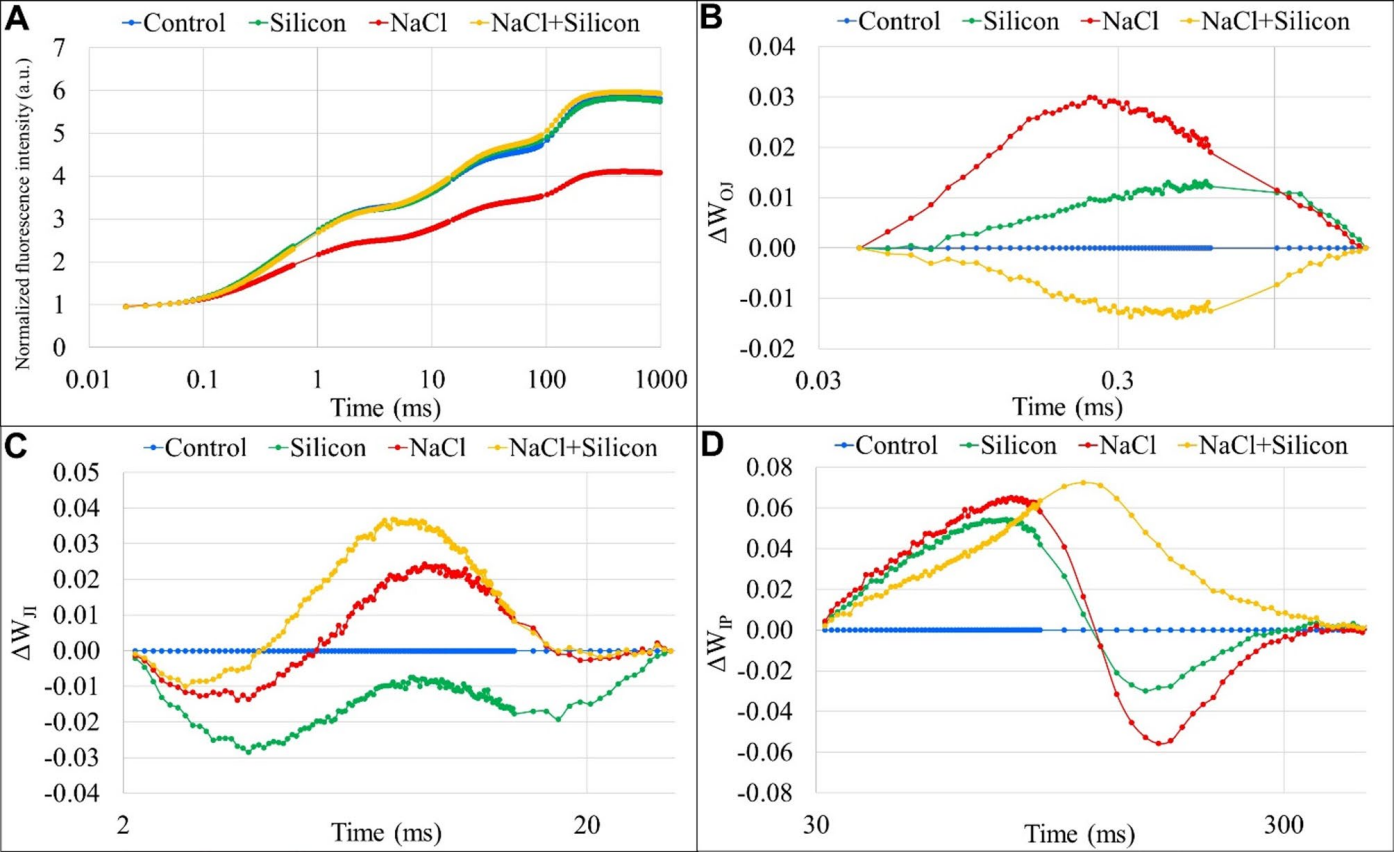
Effect of foliar application silicon on the fast chlorophyll *a* fluorescence transient kinetics of buckwheat (*Fagopyrum esculentum* cv. Smuga) under NaCl stress. **(A)** Normalized fluorescence (F_t_/F_O_); **(B)** Variable changes in the shape of prompt fluorescence transient curves, expressed as: ΔW_OJ_ = [(F_t_−F_O_)/(F_J_−F_O_)], reflecting the O to J phase; **(C)** ΔW_JI_ = [(F_t_−F_J_)/(F_I_−F_J_)], capturing the J to I transition; and **(D)** ΔW_IP_ = [(F_t_−F_I_)/(F_P_−F_I_)], representing the I to P phase. Variations between phases are shown by subtracting control group dynamics from those of the treatment groups. Data points represent mean values for each treatment group (*n* = 5). Treatments: Control (no NaCl or Si application), Silicon (foliar application of 1 mM Si without NaCl), NaCl (50 mM NaCl treatment without Si application), and NaCl + Silicon (foliar application of Si under NaCl stress).

The influence of foliar-applied Si on the donor and acceptor sides of PSII under saline and non-saline conditions was investigated using fast chlorophyll *a* fluorescence (OJIP) kinetics. The normalized fluorescence transient, calculated as F_t_/F_O_, between the O to P phases, is presented in Fig. 3A to illustrate the polyphasic processes of the OJIP curves. This polyphasic analysis revealed that NaCl stress caused reductions in the O-J, J-I, and I-P phases compared to control plants. In contrast, Si-treated plants exposed to salt stress displayed no significant changes in the O-J and J-I phases; however, the I-P phase exhibited a marked increase in fluorescence intensity relative to control plants (Fig. 3A).

Salt stress led to the emergence of a positive K band between 0.2 and 0.3 ms in the ΔW_OJ_ phase. In contrast, applying foliar Si under NaCl stress effectively suppressed this K band (Fig. 3B). Under NaCl stress, the relative changes in the ΔW_JI_ phase initially showed negative values but became positive as stress progressed. A similar trend was observed in Si-treated plants under NaCl stress, although these plants exhibited a more pronounced positive peak compared to those experiencing NaCl stress alone (Fig. 3C). In the ΔW_IP_ phase, fluorescence dropped sharply after 80 ms, becoming negative under salt stress. Conversely, the NaCl + Silicon treatment exhibited a positive peak (Fig. 3D).

**Fig. 4:**
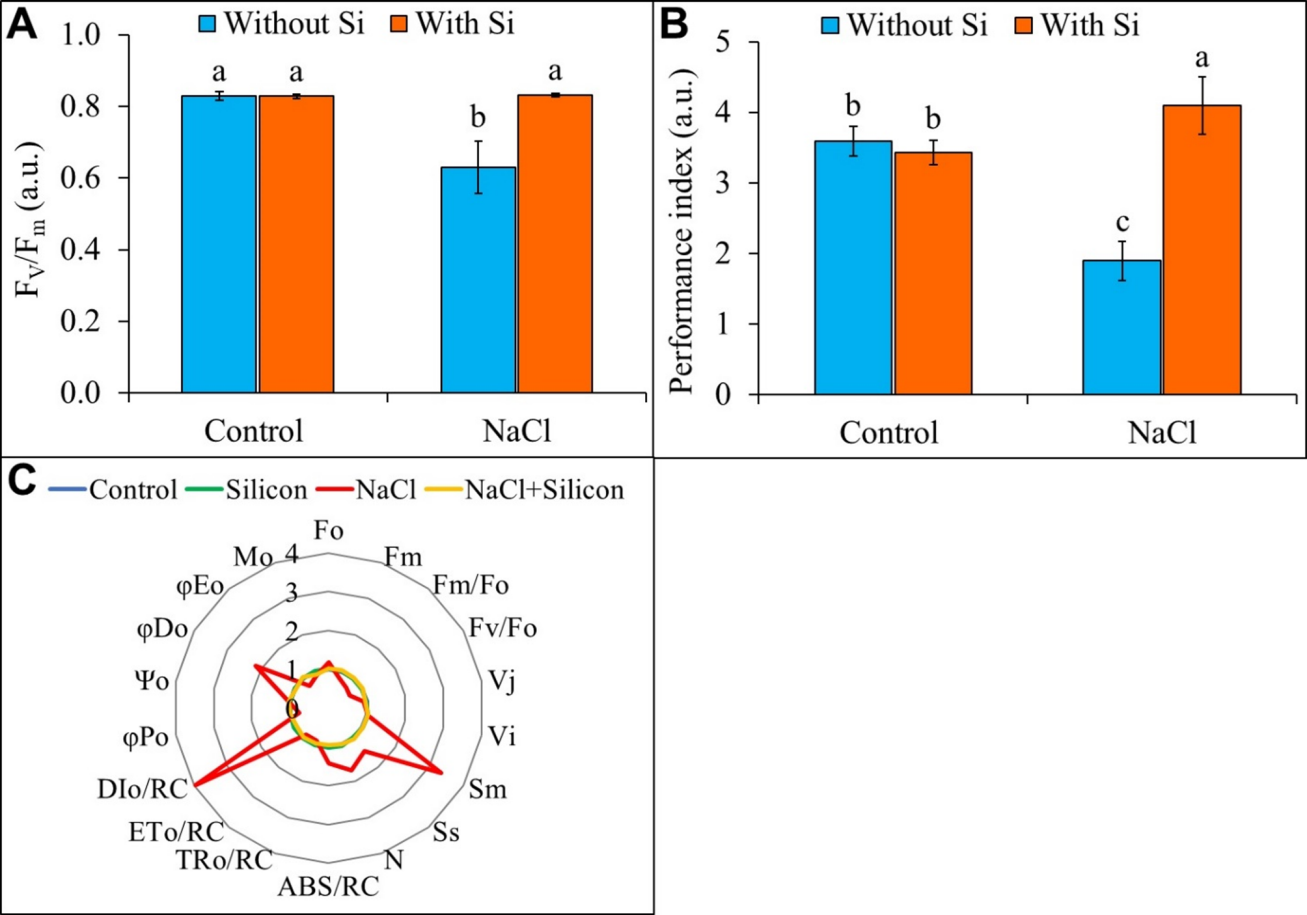
Fast chlorophyll *a* fluorescence kinetics (OJIP) in dark-adapted buckwheat leaves under different treatment conditions. **(A)** Maximum photochemical efficiency of PSII (F_V_/F_m_) and **(B)** performance index (PI_abs_). Data are presented as mean ± SD (*n* = 5), different letters indicate significant differences among treatment groups after applying a Tukey’s HSD test at *p* ≤ 0.05. **(C)** Spider plots displaying JIP parameters, calculated from chlorophyll *a* fluorescence transient curves, in salt-stressed buckwheat plants (*Fagopyrum esculentum* cv. Smuga), with or without Si application, normalized to the control. Treatments: Control (no NaCl or Si application), Silicon (foliar application of 1 mM Si without NaCl), NaCl (50 mM NaCl treatment without Si application), and NaCl + Silicon (foliar application of Si under NaCl stress).

Under NaCl stress, the F_V_/F_m_ was decreased by 24% compared to the control. However, foliar-applied Si under NaCl stress mitigated this reduction, improving F_V_/F_m_ by 32% relative to NaCl-stressed plants (Fig. 4A). A similar trend was observed in the performance index (PI_abs_) value, which was reduced by 47% compared to the control. In contrast, Si application under salt stress notably boosted the PI_abs_ by 2.16-fold compared to the plants exposed to NaCl stress only (Fig. 4B).

The quantitative analysis of JIP test parameters is summarized in Table 1, with additional visualization in Fig. 4C, where a spider plot normalizes treatment data against the control group. Exposure to NaCl stress caused a significant increase in F_O_ by 18%, S_m_ by 3.36-fold, S_S_ by 44%, N by 57%, ABS/RC by 42%, DI_0_/RC by 3.96-fold, and ΦD_O_ by 2.17-fold compared to the control (Table 1). These changes reflect substantial deviations from the control values, as illustrated by the larger distances in the spider plot (Fig. 4C). Conversely, significant reductions were observed in F_m_, F_m_/F_O_, F_V_/F_O_, TR_0_/RC, ET_0_/RC, ΦP_O_, and ΦE_O_ by 17%, 30%, 37%, 11%, 10%, 24%, and 25%, respectively, under salt stress (Table 1). These alterations in parameters under NaCl stress suggest a considerable impact on the structural and functional integrity of the photosynthetic machinery, particularly in terms of energy dissipation and reaction center (RC) activity. However, when Si was applied, these NaCl-induced changes were significantly alleviated, with reductions in F_O_, S_m_, S_S_, N, ABS/RC, DI_0_/RC, and ΦD_O_ by 14%, 69%, 28%, 37%, 32%, 76%, and 55%, respectively, compared to plants under NaCl stress alone. Meanwhile, the values of F_m_, F_m_/F_O_, F_V_/F_O_, ET_0_/RC, ΦP_O_, and ΦE_O_ increased by 25%, 46%, 60%, 10%, 32%, and 38%, respectively, under similar conditions, whereas the improvement in TR_0_/RC was insignificant (Table 1). Thus, Si application under salt stress effectively modulated these parameters, bringing them closer to control values. This improvement is visually evident in the spider plot, where Si-treated plants under NaCl stress show minimal deviation from the control across most parameters (Fig. 4C). Other parameters, such as V_J_, V_I_, Ψ_O_, and M_O_, were not significantly influenced in either NaCl- or NaCl + Si-treated plants.

**Table 1:** Effect of foliar application of silicon on the chlorophyll *a* fluorescence transient parameter of buckwheat (*Fagopyrum esculentum* cv. Smuga) under NaCl stress.

Parameters	Control	Silicon	NaCl	NaCl + Silicon
F_O_	7461.4 ± 402.09^b^	7679.4 ± 618.63^b^	8819 ± 1261.09^a^	7575.4 ± 446.87^b^
F_m_	43,817 ± 1569.98^a^	44676.6 ± 2742.06^a^	36,274 ± 5877.13^b^	45190.6 ± 1816.99^a^
F_m_/F_O_	5.89 ± 0.45^a^	5.83 ± 0.21^a^	4.10 ± 0.58^b^	5.97 ± 0.15^a^
F_V_/F_O_	4.89 ± 0.45^a^	4.83 ± 0.21^a^	3.10 ± 0.58^b^	4.97 ± 0.148^a^
V_J_	0.44 ± 0.03^a^	0.44 ± 0.02^a^	0.40 ± 0.04^a^	0.42 ± 0.04^a^
V_I_	0.69 ± 0.02^a^	0.72 ± 0.04^a^	0.72 ± 0.03^a^	0.71 ± 0.04^a^
S_m_	473.25 ± 63.27^b^	477.59 ± 30.17^b^	1589.52 ± 155.07^a^	486.15 ± 24.47^b^
S_S_	0.68 ± 0.02^b^	0.67 ± 0.05^b^	0.98 ± 0.09^a^	0.71 ± 0.04^b^
N	697.21 ± 107.31^b^	716.79 ± 53.59^b^	1095.33 ± 143.20^a^	687.19 ± 41.84^b^
ABS/RC	1.77 ± 0.08^b^	1.82 ± 0.14^b^	2.52 ± 0.35^a^	1.69 ± 0.09^b^
TR_0_/RC	1.47 ± 0.05^a^	1.50 ± 0.11^a^	1.31 ± 0.11^b^	1.41 ± 0.07^ab^
ET_0_/RC	0.82 ± 0.03^a^	0.84 ± 0.05^a^	0.74 ± 0.05^b^	0.82 ± 0.06^a^
DI_0_/RC	0.30 ± 0.04^b^	0.31 ± 0.03^b^	1.20 ± 0.21^a^	0.29 ± 0.02^b^
ΦP_O_	0.832 ± 0.01^a^	0.829 ± 0.01^a^	0.630 ± 0.09^b^	0.828 ± 0.004^a^
Ψ_O_	0.558 ± 0.03^a^	0.559 ± 0.02^a^	0.597 ± 0.03^a^	0.577 ± 0.04^a^
ΦD_O_	0.17 ± 0.018^b^	0.17 ± 0.006^b^	0.37 ± 0.017^a^	0.17 ± 0.004^b^
ΦE_O_	0.46 ± 0.03^a^	0.46 ± 0.02^a^	0.35 ± 0.05^b^	0.48 ± 0.04^a^
M_O_	0.65 ± 0.06^a^	0.66 ± 0.07^a^	0.58 ± 0.10^a^	0.59 ± 0.08^a^

Values are presented as mean ± SD (*n* = 5); different letters indicate significant differences among treatment groups after applying a Tukey’s HSD test at *p* ≤ 0.05. Treatments: Control (no NaCl or Si application), Silicon (foliar application of 1 mM Si without NaCl), NaCl (50 mM NaCl treatment without Si application), and NaCl + Silicon (foliar application of Si under NaCl stress).

**Fig. 5:**
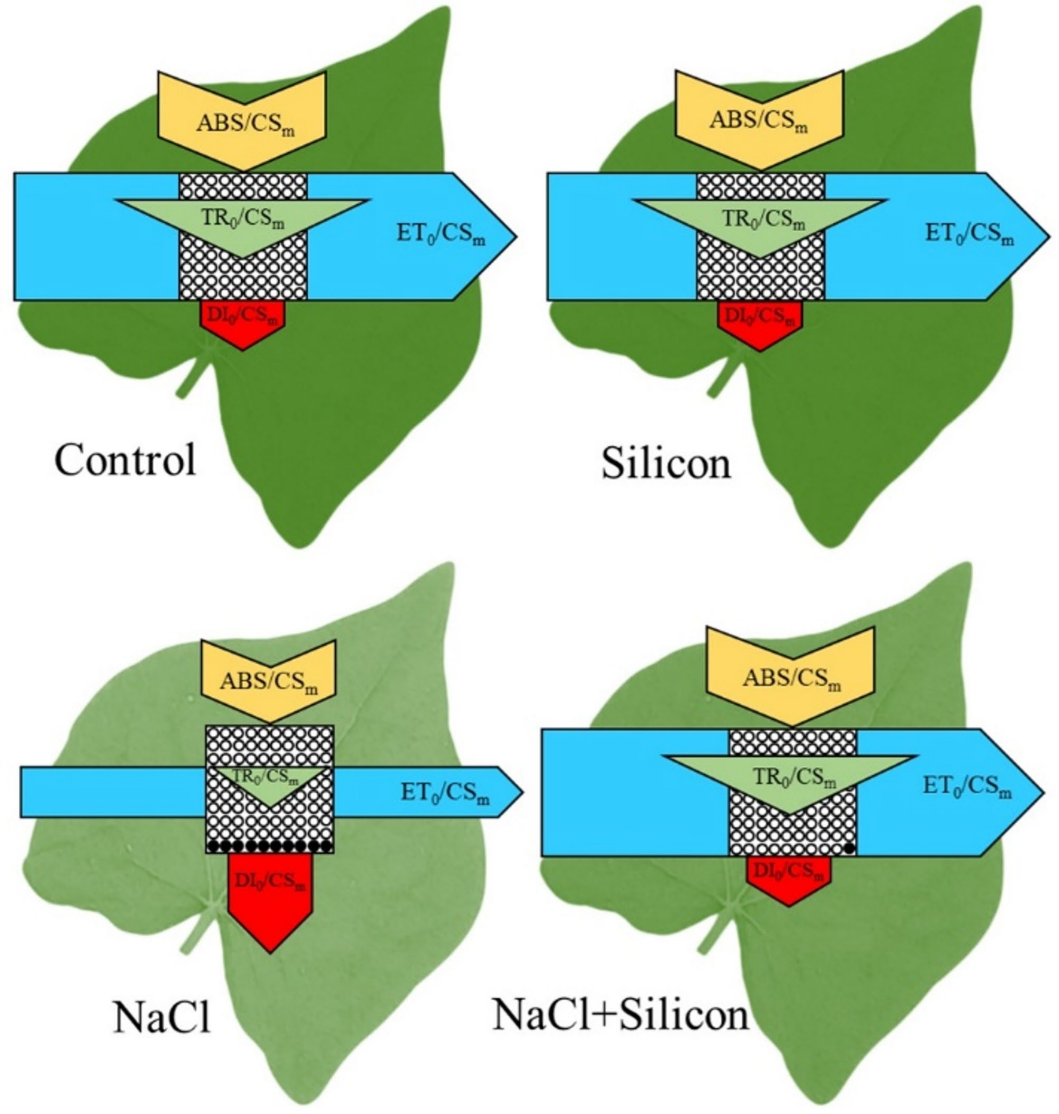
Energy pipeline model of phenomenological fluxes of buckwheat (*Fagopyrum esculentum* cv. Smuga) under different treatment conditions. This schematic illustrates specific energy fluxes per cross-section (CS_m_) for photosystem II, including absorption (ABS/CS_m_), trapping (TR_0_/CS_m_), electron transport (ET_0_/CS_m_), and dissipation (DI_0_/CS_m_). Each flux is represented as a distinct component within the photosystem II membrane model, shown as embedded protein units (boxes) to highlight energy partitioning under different treatment conditions. The varying widths of the boxes represent the relative magnitude of each energy flux per cross-section. Active reaction centers are depicted as open circles, while inactive reaction centers are represented as filled circles. Treatments: Control (no NaCl or Si application), Silicon (foliar application of 1 mM Si without NaCl), NaCl (50 mM NaCl treatment without Si application), and NaCl + Silicon (foliar application of Si under NaCl stress).

The phenomenological energy flux model across different treatment groups has been illustrated in Fig. 5, providing a detailed perspective on photosystem performance and insights into the response of RCs under light exposure. Salt stress led to a decrease in the ABS/CS_m_, TR_0_/CS_m_, and ET_0_/CS_m_, while DI_0_/CS_m_ increased compared to the control group. In contrast, plants treated with both NaCl and Si exhibited levels of ABS/CS_m_, TR_0_/CS_m_, ET_0_/CS_m_, and DI_0_/CS_m_ similar to those of the controls, highlighting the positive role of Si in maintaining photosystem function under salt stress. Furthermore, salt stress resulted in an increase in the number of inactive RCs compared to the control. However, plants treated with NaCl and Si showed a significant increase in the number of active RCs compared to those subjected to NaCl stress alone (Fig. 5).

### Effect of silicon on the photosynthetic performance of buckwheat plants under salt stress

**Fig. 6:**
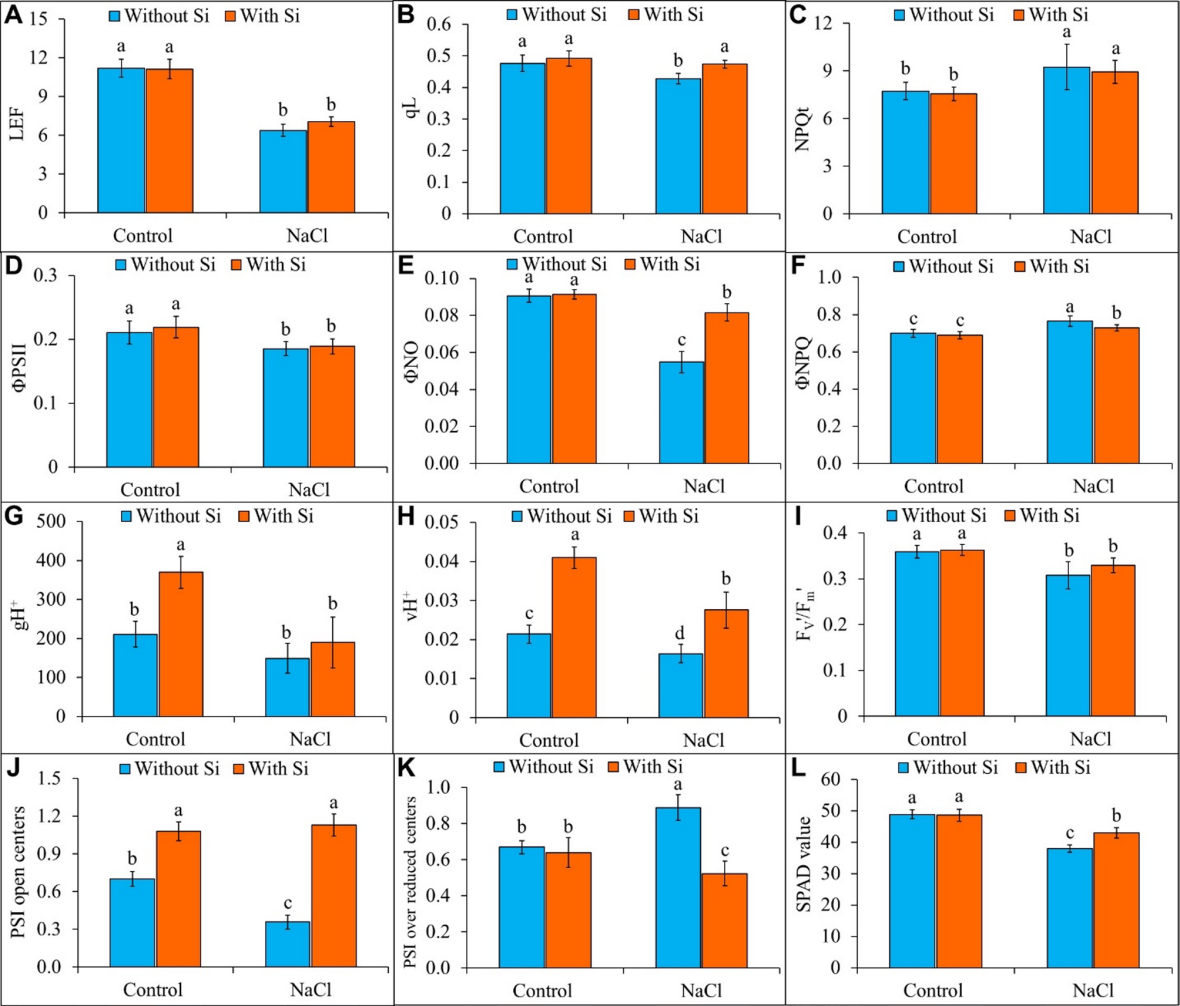
Effect of foliar application of silicon on the photosynthetic attributes, **(A)** linear electron flow (LEF), **(B)** photochemical quenching of variable fluorescence (qL), **(C)** total non-photochemical quenching of maximum fluorescence (NPQt), **(D)** effective quantum yield of PSII (ΦPSII), **(E)** quantum yield of light-independent energy dissipation in PSII (ΦNO), **(F)** quantum yield of light-induced energy dissipation in PSII (ΦNPQ), **(G)** proton conductance of the thylakoid membrane (gH^+^), **(H)** steady-state proton flux (vH^+^), **(I)** quantum yield of light-adapted PSII (F_V_´/F_m_´), **(J)** PSI open centers, **(K)** PSI over reduced centers, and **(L)** SPAD value of buckwheat (*Fagopyrum esculentum* cv. Smuga) under NaCl stress. Data are presented as mean ± SD (*n* = 5); different letters indicate significant differences among treatment groups after applying a Tukey’s HSD test at *p* ≤ 0.05. Treatments: Control (no NaCl or Si application), Silicon (foliar application of 1 mM Si without NaCl), NaCl (50 mM NaCl treatment without Si application), and NaCl + Silicon (foliar application of Si under NaCl stress).

Salt stress significantly impacted several photosynthetic parameters, leading to notable declines in key metrics compared to control plants. Specifically, salt stress caused a 43% reduction in LEF (Fig. 6A), a 10% decrease in qL (Fig. 6B), a 12% reduction in ΦPSII (Fig. 6D), a 40% decrease in ΦNO (Fig. 6E), a 23% decrease in vH^+^ (Fig. 6H), a 14% drop in F_V_′/F_m_′ (Fig. 6I), a 49% reduction in the number of open centers in photosystem I (PSI; Fig. 6J)), and a 22% decline in SPAD values (Fig. 6L). These changes indicate overall stress on the photosynthetic system. Conversely, salt stress increased NPQt by 20% (Fig. 6C), ΦNPQ by 9% (Fig. 6F), and an over-reduction of PSI centers by 33% (Fig. 6K). These adaptations reflect a shift toward protective energy dissipation in response to stress. However, the application of foliar Si substantially mitigated these stress effects, positively affecting photosynthetic attributes under light-adapted conditions. Silicon treatment increased qL by 11% (Fig. 6B), ΦNO by 49% (Fig. 6E), vH^+^ by 68% (Fig. 6H), PSI open centers by 215% (Fig. 6J), and SPAD value by 13% (Fig. 6L) compared to NaCl stress alone, suggesting improved energy utilization and a reduction in excess excitation energy in the photosystems. Additionally, Si-treated plants under salt stress showed a 5% decrease in ΦNPQ (Fig. 6F) and a 41% reduction in over-reduced PSI centers (Fig. 6K), highlighting Si’s role in alleviating PSI stress and optimizing energy distribution within the photosynthetic apparatus.

## Discussion

Salinity is a major abiotic stressor that significantly disrupts plant physiological and biochemical processes, ultimately impairing growth, development, and yield^4^. The detrimental effects of salinity arise primarily from hyperosmotic and hyperionic conditions, which compromise key metabolic pathways such as photosynthesis, osmotic and ionic balance, and ROS regulation. This disruption leads to excessive ROS production, ultimately resulting in stunted plant growth and development^19^. The present study revealed that salt stress significantly inhibited plant growth by reducing root and shoot lengths, leaf thickness, and overall biomass. This growth inhibition is primarily attributed to excessive ion accumulation in plant cells coupled with reduced uptake of essential nutrients and water, which together restrict cell division and development^20^. Similar reductions in biomass and shorter shoots and roots under salt stress have been reported in various plant species, including maize^21^, barley^22,23^, wheat^24^, and black gram^25^. However, Si application reversed the toxic effects of salt-induced damage, resulting in a significant increase in growth-related attributes, including root length, leaf thickness, and dry mass. Silicon mitigates salt stress by inhibiting excessive Na^+^ uptake in roots and reducing its translocation to leaves through the transpiration stream. It also induces suberization of the exodermis and endodermis, thereby limiting Na^+^ toxicity while enhancing nutrient uptake, which in turn promotes plant growth^26^. Furthermore, under stress conditions, Si improves plant development by restoring cell membrane integrity and providing defense against tissue damage^27^. With Si supplementation, plants require less water and are better able to withstand salt-induced osmotic stress^25^. Application of Si to NaCl-treated plants increased leaf thickness in this study, likely due to Si deposition in cell walls that forms a double-layered cuticle in the epidermal layer^10,28^. Moreover, Si has been shown to regulate phytohormones, such as gibberellins and jasmonic acid, which play pivotal roles in stress response mechanisms and sustain plant growth^29,30^. Therefore, the current study’s findings corroborate previous research on cotton^14^, barley^23^, and black gram^25^ in which Si application significantly improved plant growth under salt stress.

Optimal plant growth is intricately linked to photosynthetic efficiency, which supplies the energy required for development^31^. Chlorophyll, an essential photosynthetic pigment, plays a central role in light absorption, energy transformation, and carbon fixation. When the rate of light absorption by photosynthetic pigments surpasses that of energy utilization, photoinhibition is accelerated, further impairing photosynthetic capacity^32^. In this study, NaCl stress resulted in lower SPAD values—a proxy of Chl content—which corresponded with diminished photosynthetic efficiency and reduced biomass production. These findings are consistent with previous reports demonstrating that salt stress decreases Chl content, photosynthetic performance, and overall plant growth^14,33^. Notably, the application of Si mitigated the adverse effects of salt stress on SPAD values, similar improvements in SPAD values have been observed in wheat subjected to Si treatment under stress conditions^34 ^further corroborating our findings. This study further revealed that NaCl-induced impairment of photosynthetic efficiency is evidenced by alterations in chlorophyll *a* fluorescence transient parameter. The emergence of a positive K-band under salt stress indicated damage to the OEC and PSII^35,36^. The damage leads to impaired thylakoid membrane integrity and inefficient electron transport at the PSII donor site^35,36^. Such a positive K-band appearance under salt stress has been reported in cotton^14^ and barley^23^ corroborating our current finding. However, the reduced intensity of K-bands in Si-treated plants suggests that the OEC is stabilized, thylakoid membrane integrity is preserved, and PSII functionality is maintained. This suppression of the K-band with Si application was also reported by Akhter et al.^23^ in barley under similar conditions. Additionally, the accumulation of inactive RCs in PSII further diminishes electron acceptance from the water-splitting complex, resulting in a decrease in the maximum quantum yield of PSII (F_V_/F_m_)^37^. This dysfunction compromises electron transport, lowers photochemical activity, and disrupts overall PSII function, as evidenced by the reduced F_V_/F_m_ and PI_abs_ in the current study. Previous findings similarly indicate that salt stress disrupts PSII functions by reducing key photosynthetic indicators such as F_V_/F_m_ and PI_abs_ in cotton^14^ and oat plants^38^. Performance index (PI_abs_), a comprehensive index for assessing photosynthetic performance, integrates parameters including ABS/RC, ΦP_O_, and ΦE_O_, thereby evaluating the impact of stress on the photosynthetic apparatus^37^. Under NaCl exposure, reductions in ΦP_O_ and ΦE_O_, combined with an increase in ABS/RC, lead to impaired energy transfer from antenna pigments to RCs, hindering electron flow within PSII. The blockage of electron transport in the ETC can cause photooxidation of thylakoid membrane proteins. In response, plants adjust their photosynthetic machinery to dissipate excess energy (DI_0_/RC), thereby preventing further photodamage^39^; accordingly, DI_0_/RC values of our study were notably higher in the stressed plants. Similar increases of DI_0_/RC under salt stress have been reported in cotton^14^ and oats^38^. Conversely, Si supplementation reduced DI_0_/RC and improved ΦP_O_ and ΦE_O_ in the salt-stress cotton plants, indicating more efficient electron transfer from antennae to RCs^14^. Akhter et al.^23^ also reported a similar response with Si application under salt stress in barley, where ΦP_O_ was improved in Si-treated plants under stress^39^. Damage to PSII under salt stress is also indicated by reduced F_V_/F_O_ and F_m_/F_O_ ratios, key indicators of physiological stress^40^. The declines in these ratios in our study suggest impairments in water splitting and electron transport within PSII, leading to inefficient energy transfer^14,23^. Similar reductions of F_V_/F_O_ and F_m_/F_O_ ratios have been reported by Akhter et al.^23^; in contrast, Si-treated plants showed improved ratios, indicating enhanced water splitting and electron transport within PSII^23^. This finding also aligns with previous reports of Ghassemi-Golezani^41^ in mung bean and Zhang et al.^42^ in tomato, which demonstrated that Si supplementation improves F_V_/F_O_ under stress. Additionally, NaCl stress significantly increased S_m_, S_S_, and N levels in plants compared to the control, indicating enhanced electron transport through Q_A_ reduction and increased energy fluxes associated with chlorophyll fluorescence transients. These changes likely reflect the plant’s physiological adjustments to cope with stress and meet the elevated energy demands^43^. Conversely, foliar-applied Si notably decreased these parameters, suggesting its role in stabilizing electron transfer processes and restoring balance to chlorophyll fluorescence transients^14^. The phenomenological energy flux model identified PSII as a critical site of stress sensitivity, revealing reductions in ABS/CS_m_, TR_0_/CS_m_, and ET_0_/CS_m_ under salt stress compared to controls. This reduction is likely due to the conversion of active RCs into inactive ones, thereby impairing energy trapping and electron transport in PSII^44^. Similar results were reported in salt-stressed tomato leaves by Chen et al.^44^demonstrating the universal impact of salt stress on photosynthetic machinery. However, foliar-applied Si effectively mitigated these detrimental effects by enhancing energy transfer and decreasing energy dissipation^23^. Silicon enhances the activities of key enzymes such as ferredoxin-NADP reeducate, ATP synthase, and ribulose-1, 5-bisphosphate carboxylase/oxygenase (RubisCO), and upregulates RubisCO-related genes, thereby improving photosynthesis under stress^14^. It also positively influences light-adapted photosynthetic parameters, increasing photochemical quenching and regulating energy dissipation. By reducing non-photochemical quenching and preventing over-reduction of PSI centers, Si optimizes energy capture while minimizing photoinhibition^32^. Song et al.^12^ observed enhanced photosynthesis in the Si-treated rice due to increased mesophyll conductance and improved chloroplast structure, while Habibi and Hajiboland^45^ and Muneer et al.^46^ reported enhanced PSII efficiency and reduced photoinhibition in the Si-treated plants under stress. Ahmad et al.^47^ demonstrated similar effects in salt-stressed mung bean, where Si supplementation increased ΦPSII, ΦNPQ, and F_V_/F_m_, further corroborating its role in alleviating stress-induced photochemical inefficiencies. Silicon application effectively reduces stress-induced damage by stabilizing the photosynthetic apparatus, protecting PSI and PSII, and maintaining efficient electron transport^47^. By enhancing Chl content, preserving thylakoid membrane integrity, and optimizing energy fluxes, Si promotes resilience in photosynthesis and mitigates salt-induced damage. Therefore, these findings highlight the protective role of Si in improving plant stress tolerance and sustaining productivity under saline conditions.

## Materials and methods

### Plant material and experimental conditions

Buckwheat achenes (*F. esculentum* Moench cv. Smuga) were utilized as study material, collected from Logistic sp. z o.o., Góra, Poland. The experiment was conducted during the growing season of 2024 at Poznań University of Life Sciences, Poznań, Poland. The study followed a completely randomized design with five replicates (*n* = 5). The daily photosynthetic photon flux density (PPFD) and the temperature during the growth period have been included in the supplementary file **(Supplementary Fig. 1)**. The growth medium comprised 200 g of deacidified peat (pH 6.5). Uniform, mature achenes were hand-sorted and thoroughly rinsed multiple times with distilled water (dH_2_O). Four achenes were sown per plastic pot (9 cm × 9 cm width-height), and seven days after sowing (DAS), seedlings were thinned to retain one healthy and uniform-sized seedling per pot. Two treatment groups were established from 21 DAS: one group was irrigated with dH_2_O, and a second group was exposed to NaCl stress via irrigation with 50 mM NaCl solution. At the same time, a foliar application of Si was performed using a 1 mM solution of sodium metasilicate nonahydrate (Na_2_SiO_3_·9H_2_O) at a dose equivalent to 400 L ha^− 1^. The dose of each plant was calculated based on the planting density of 150 plants per square meter. This foliar spray was applied twice, at 21 DAS and again at 24 DAS. To ensure consistent conditions, control plants and NaCl-stressed plants without Si treatment received a foliar spray of dH_2_O only. The treatment groups for the study were as follows: (i) Control, well-watered with foliar application of dH_2_O only (no Si); (ii) Silicon, well-watered with foliar application of 1 mM Si; (iii) NaCl, irrigated with 50 mM NaCl and foliar application of dH_2_O only (no Si); and (iv) NaCl + Silicon, irrigated with 50 mM NaCl and foliar application of 1 mM Si. Morphological and photosynthetic attributes were measured seven days after treatment exposure.

### Fast chlorophyll *a* fluorescence kinetics (OJIP) measurement

Chlorophyll *a* fluorescence transient was measured from the leaves at the third oldest leaf of each plant using a FluorPen FP 110/D (Photon System Instruments, Drasov, Czech Republic) with detachable dark adaptation clips. Leaves were dark-adapted for 25 min before measurement using the OJIP protocol provided by the manufacturer. The light intensities of flash pulse, super pulse, and actinic pulse were set to 30%, 80%, and 60%, respectively, with a wavelength of 455 nm. The chlorophyll *a* fluorescence transient (F_t_) was normalized against the minimal fluorescence (F_O_), expressed as F_t_/F_O_, and plotted on a logarithmic time scale. The ratio of variable fluorescence was calculated as the difference between the variable fluorescence (V_t_) of the treatment and control conditions relative to the amplitude of F_J_–F_O_ (ΔW_OJ_), F_I_–F_J_ (ΔW_IJ_), and F_P_–F_I_ (ΔW_IP_). Parameters derived from the fluorescence measurements are summarized in Table 2. In addition, photosynthetic parameters based on fluorescence and electrochromic shifts were evaluated using a MultispeQ V2.0 device (PhotosynQ Inc., East Lancing, MI, USA) connected to the PhotosynQ platform (http://www.photosynq.org). Linear electron flow (LEF), photochemical quenching of variable fluorescence (qL), total non-photochemical quenching of maximal fluorescence (NPQt), the effective quantum yield of PSII (ΦPSII), the quantum yield of light-independent energy dissipation in PSII (ΦNO), the quantum yield of light-induced energy dissipation in PSII (ΦNPQ), proton conductance of the thylakoid membrane (gH^+^), steady-state proton flux (vH^+^), the quantum yield of light-adapted PSII (F_V_ʹ/F_m_ʹ), PSI open centers, PSI over reduced centers, and SPAD value were driven from the MultispeQ V2.0 device^48^.

**Table 2:** A summary of parameters and the equations used to derive data from chlorophyll *a* fluorescence transient is provided.

Parameters	Explanations
F_t_	Fluorescence at time t when the actinic light illumination was turned on
F_J_ = F_2ms_	Fluorescence at the J-step (2 ms) of O-J-I-P transient
F_I_ = F_30ms_	Fluorescence at the I-step (30 ms) of O-J-I-P transient
F_P_ = F_m_	Maximal recorded fluorescence at peak P of O-J-I-P transient
^t^F_m_	^t^F_m_ time (in ms) to reach maximal fluorescence F_m_
Area	Total complementary area between fluorescence induction curve and F = F_m_
F_O_	Minimal fluorescence when all the reaction centers (RC) of photosystem II (PSII) are open
F_V_/F_O_	Maximum efficiency of the water diffusion reaction on the donor side of PSII
F_m_/F_O_	Maximum fluorescence normalized by minimum fluorescence
F_V_/F_m_	Maximum photochemical efficiency of PSII
V_J_ = (F_J_−F_O_)/(F_m_−F_O_)	Relative variable fluorescence at the step J after 2 ms
V_I_ = (F_I_−F_O_)/(F_m_−F_O_)	Relative variable fluorescence at the step I after 30 ms
M_O_ = (ΔV/Δt)_0_ = 4(F_300µs_−F_O_)/(F_m_−F_O_)	Approximated initial slope (per ms) of the fluorescence transient
S_m_ = Area/(F_m_−F_O_)	Normalized total complementary area above the O-J-I-P transient
S_S_ = V_J_/M_O_	Normalized total complementary area corresponding only to the O-J phase
N = S_m_/S_S_ = S_m_M_O_(1/V_J_)	Turnover number: number of Q_A_ reduction events that occurred between time 0 and ^t^F_m_
ABS/RC = M_O_(1/V_J_)(1/ΦP_O_)	Absorption flux per RC at time 0
TR_0_/RC = M_O_(1/V_J_)	Energy flux trapped per active RC at time 0
ET_0_/RC = M_O_(1/V_J_) Ψ_0_	Rate of electron transport per active RC at time 0
DI_0_/RC = (ABS/RC)–(TR_0_/RC)	Energy flux not intercepted per RC at time 0
ΦP_O_ = TR_0_/ABS = [1–(F_O_/F_m_)]	Maximum quantum yield of primary photochemistry
Ψo = ET_0_/TR_0_ = (1–V_J_)	Probability of electron transport outside Q_A_^−^ at t = 0
ΦE_O_ = ET_0_/ABS = [1–(F_O_/F_m_)]Ψ_O_	Quantum yield of electron transport (at t = 0)
ΦD_O_ = 1–ΦP_O_ = (F_O_/F_m_)	Quantum yield of energy dissipation (at t = 0)
PI_abs_ = $\:\frac{\text{R}\text{C}}{\text{A}\text{B}\text{S}}\:.\frac{{\Phi\:}\text{P}\text{O}}{1-\:{\Phi\:}\text{P}\text{O}}.\:\frac{{\Psi\:}\text{o}}{1-\:{\Psi\:}\text{o}}$	Performance index of PSII on absorption basis
ABS/CS_m_ ≈F_m_	Absorption flux per cross-section, approximated by F_m_
TR_0_/CS_m_ = ΦP_O_(ABS/CS_m_)	Trapped energy flux per cross-section at t = 0
ET_0_/CS_m_ = ΦE_O_(ABS/CS_m_)	Electron transport flux per cross-section at t = 0
DI_0_/CS_m_ = (ABS/CS_m_)–(TR_0_/CS_m_)	Dissipated energy flux per cross-section at t = 0

OJIP transient parameters were calculated following the JIP test protocols as outlined by Strasser et al.^49^ and Kalaji et al.^50^.

### Measurement of plant growth attributes

After measuring chlorophyll *a* fluorescence, plants from each treatment group were carefully uprooted and thoroughly rinsed with running tap water to remove soil and debris from the roots. Roots were then separated from the shoots, and the lengths of both roots and shoots were measured using a ruler. Stem diameter and leaf thickness were measured with a slide calipers, and the number of leaves and primary branches was counted manually for each plant. Fresh weight (FW) was determined by weighing each plant on an electronic balance. Plants were then oven-dried at 70 °C until fully dried, after which dry weight (DW) was recorded.

### Statistical analysis

Raw data were initially visualized using Microsoft Excel (Microsoft Corporation, Redmond, Washington, USA). Measurements exhibiting stable low fluorescence, indicative of closed dark-adaptation clips, or any erroneous measurements were excluded before statistical analysis. Data are presented as the mean ± standard deviation of five plants (*n* = 5). All data were analyzed using a one-way analysis of variance (ANOVA), with means compared using Tukey’s honestly significant difference (HSD) test at a 5% significance level. Analyses were performed in RStudio version 4.4.1 (RStudio Inc., Boston, MA, USA).

## Conclusions

In this study, salt stress resulted in substantial disruptions in the photosynthetic machinery, reflected by altered chlorophyll *a* fluorescence transient parameter, and the emergence of a positive K-band, indicative of OEC damage and compromised PSII functionality of buckwheat. As a result, photochemical efficiency, electron transport, energy utilization, and plant growth all declined. However, foliar-applied Si alleviated these detrimental effects by stabilizing the thylakoid membrane, reducing non-photochemical quenching, and improving photochemical energy flux. Silicon-treated plants showed enhanced F_V_/F_m_, PI_abs_, and ET_0_/RC, while mitigating DI_0_/RC. Furthermore, Si improved Chl content and the balance between PSI and PSII activities, as evidenced by an increase in qL, open PSI centers, and ΦE_O_. Moreover, the application of Si improved morphological attributes such as root length, stem diameter, leaf thickness, and biomass accumulation in plants under salt stress, further emphasizing Si’s role in promoting growth under stress conditions. This research highlights the potential of Si as an effective tool for improving crop resilience to salt stress. However, future studies should focus on elucidating the molecular and antioxidant mechanisms influenced by Si under salinity stress in buckwheat, providing deeper insights into its protective role at the biochemical and genetic levels.

## Electronic supplementary material

Below is the link to the electronic supplementary material.

Supplementary Material 1

## Data Availability

The raw data related to the article has been deposited in the Open Data Repository RepOD (https://repod.icm.edu.pl/https://repod.icm.edu.pl/)- the PULS institutional repository with DOI: 10.18150/ASKKCB.
