# Supplementary material for: Silicon-induced photosynthetic adaptations in common buckwheat under salt stress revealed by prompt chlorophyll a fluorescence analysis
Source: Sci Rep. 2025 Jun 2;15:19343. doi: 10.1038/s41598-025-04159-1 (PMC12130291; doi:10.1038/s41598-025-04159-1)
Supplement: Supplementary file 1 — Supplementary Material 1 [file 41598_2025_4159_MOESM1_ESM.docx]

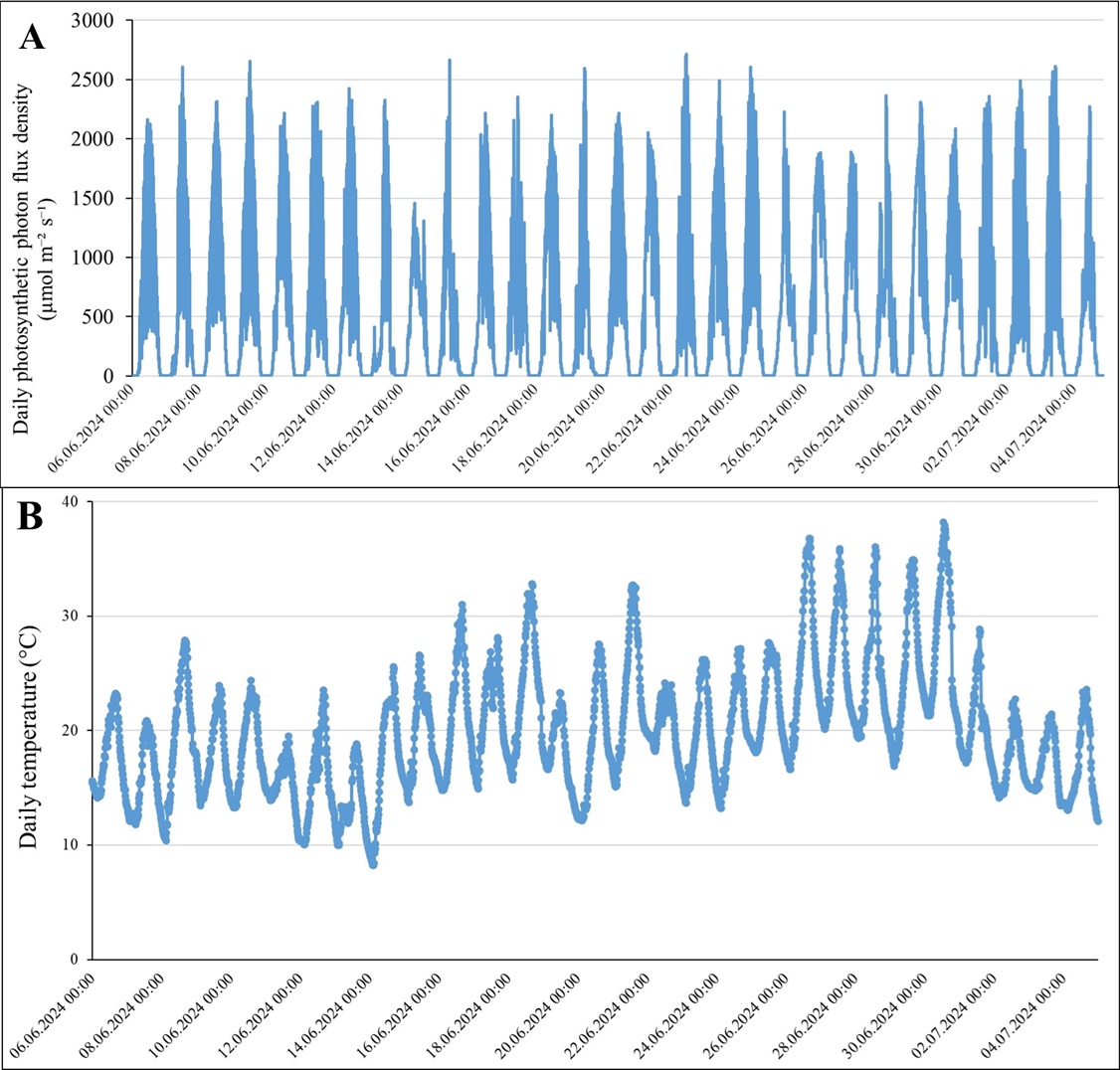


**Supplementary Figure 1.** **(A)** Daily photosynthetic photon flux density and **(B)** temperature recorded throughout the experimental period.
